# Vitamin-D status in sixty adolescents with idiopathic scoliosis: An observational study

**DOI:** 10.12669/pjms.42.5.14121

**Published:** 2026-05

**Authors:** Xiufeng Wang, Luping Ji, Xi Wang, Jinhui Zhou

**Affiliations:** 1 Xiufeng Wang, Department of Pediatric Rehabilitation, Xingtai People’s Hospital Affiliated to Hebei Medical University, Xingtai 054000, Hebei, China; 2 Luping Ji, Department of Pediatric Rehabilitation, Xingtai People’s Hospital Affiliated to Hebei Medical University, Xingtai 054000, Hebei, China; 3 Xi Wang, Department of Pediatric Rehabilitation, Xingtai People’s Hospital Affiliated to Hebei Medical University, Xingtai 054000, Hebei, China; 4 Jinhui Zhou, Department of Pediatric Rehabilitation, Xingtai People’s Hospital Affiliated to Hebei Medical University, Xingtai 054000, Hebei, China

**Keywords:** Adolescent, Scoliosis, Vitamin-D

## Abstract

**Objectives:**

To investigate serum Vitamin-D levels in adolescents with idiopathic scoliosis and analyze the association between Vitamin-D levels and scoliosis severity.

**Methodology:**

This was a retrospective study. A total of 60 adolescents diagnosed with idiopathic scoliosis at Xingtai People’s Hospital from September 2020 to October 2024 were enrolled for observation. Serum 25-hydroxyVitamin-D(25[OH]D) concentrations were measured, and the Cobb angle of the primary curve was determined from standing spinal radiographs. Patients were stratified into two groups according to Vitamin-D levels: the observation group (insufficient Vitamin-D) and the control group (sufficient Vitamin-D). Statistical analyses were performed using the software Prism, with independent t-tests and correlation analyses.

**Results:**

Among patients with Vitamin-D deficiency or insufficiency (25[OH]D < 50 nmol/L), 11 exhibited a Cobb angle > 15°, whereas only two patients with sufficient Vitamin-D levels (25[OH]D 50–250 nmol/L) had a Cobb angle > 15°. A significant negative correlation was observed between serum 25(OH)D concentration and scoliosis severity (*P=* 0.0154 < 0.05).

**Conclusion:**

Vitamin-D deficiency may be associated with the development of adolescent idiopathic scoliosis. Adequate Vitamin-D supplementation could help mitigate curve progression and reduce scoliosis severity.

## INTRODUCTION

Adolescent idiopathic scoliosis(AIS) is defined as a lateral curvature of the spine in the coronal plane exceeding 10°, occurring during the pubertal growth period between 10 and 18 years of age.^1^,^2^ AIS represents a three-dimensional deformity involving the coronal, sagittal, and axial planes. Unlike congenital or neuromuscular scoliosis, its etiology remains largely unclear.^3^,^4^ AIS is the most common type of scoliosis, with a reported prevalence of approximately 0.47%–5.20%, and it occurs more frequently in females.^5^,^6^

The development of scoliosis is believed to be multifactorial, with contributions from somatic growth, hormonal regulation, gravity, genetic predisposition, neurological dysfunction, environmental stressors, biomechanical imbalance, and lifestyle factors.^7^-^9^ Adequate Vitamin-D status is essential for musculoskeletal development and the maintenance of normal physiological functions. Beyond its established role in bone health, Vitamin-D has been increasingly recognized as a hormone with wide-ranging biological effects across multiple organ systems, mediated through endocrine, autocrine, and paracrine mechanisms.^10^,^11^ In this study, sixty individuals with AIS were randomly selected to analyze their serum 25-hydroxyVitamin-D (25[OH]D) levels and investigate the potential association between Vitamin-D status and AIS severity, aiming to provide new insights into the etiological mechanisms of scoliosis and offer a basis for preventive and therapeutic strategies.

## METHODOLOGY

This was a retrospective study. A total of 60 adolescents diagnosed with idiopathic scoliosis at Xingtai People’s Hospital from September 2020 to October 2024 were enrolled for observation. Data were retrieved from our hospital information and management system, collect their various information of all participant.

### Ethical approval:

The study was approved by the Institutional Ethics Committee of Xingtai People’s Hospital (No.:2020[039]; Date: April 28, 2020), and written informed consent was obtained from all participants or their guardians.

### Inclusion criteria:

- Diagnosed with AIS (Cobb angle > 10°).
- Aged 6–18 years;
- Serum concentrations of 25(OH)D were measured, and the Cobb angle was determined using standing spinal radiographs.

### Exclusion criteria:

- Patients with congenital scoliosis.
- Patients with congenital metabolic or endocrine disorders.
- or those who had received Vitamin-D supplementation within the past year.

### Measurements:

The measurement of the Cobb angle was performed according to the *Practical Guidelines for Clinical Issues Related to Vitamin-D Nutrition in Chinese Children*, *Consensus on Diagnosis and Treatment of Adolescent Idiopathic Scoliosis*, and the *2016 SOSORT Guidelines: Orthopaedic and Rehabilitation Treatment of Idiopathic Scoliosis during Growth*.

Specifically, the Cobb angle was defined as the angle formed by the intersection of a line drawn along the superior endplate of the most tilted vertebra above the apex and a line drawn along the inferior endplate of the most tilted vertebra below the apex.^12^,^13^ Fasting venous blood samples were collected and analyzed in the hospital laboratory. Routine tests included fasting blood glucose, serum electrolytes, liver and renal function, and alkaline phosphatase levels to exclude patients with impaired hepatic or renal function or electrolyte disturbances. Serum 25(OH)D levels were measured using high-performance liquid chromatography.

### Data collection and Observation indicators:

Serum 25(OH)D levels were measured in 60 patients with AIS. Vitamin-D status was classified as deficiency (<30 nmol/L), insufficiency (30–50 nmol/L), sufficiency (50–250 nmol/L), and toxicity (>250 nmol/L) according to the *Practical Guidelines for Clinical Issues Related to Vitamin-D Nutrition in Chinese Children*.^11^ Due to the limited sample size, patients were stratified into two categories for analysis.

Vitamin-D sufficient group: 25(OH)D > 50–250 nmol/L; Vitamin-D insufficient group (deficient + insufficient): 25(OH)D < 50 nmol/L. Scoliosis severity was graded based on the primary Cobb angle: <10° (normal), 10°–20° (mild), 20°–45° (moderate), and >45° (severe).^14^

### Statistical analysis:

All statistical analyses were performed using SPSS 25.0. The confidence interval was 95%. Continuous data that passed the Shapiro-Wilk test were normally distributed and are presented as mean ± standard deviation (). Comparisons between groups were conducted using the independent samples t-test. Categorical data are described as frequencies and percentages (n, %), and inter-group comparisons were made using the chi-square (χ²) test. The correlation between Vitamin-D levels and Cobb’s angle was assessed by Pearson correlation analysis. A p-value<0.05 was considered statistically significant.

## RESULTS

**Fig.1:**
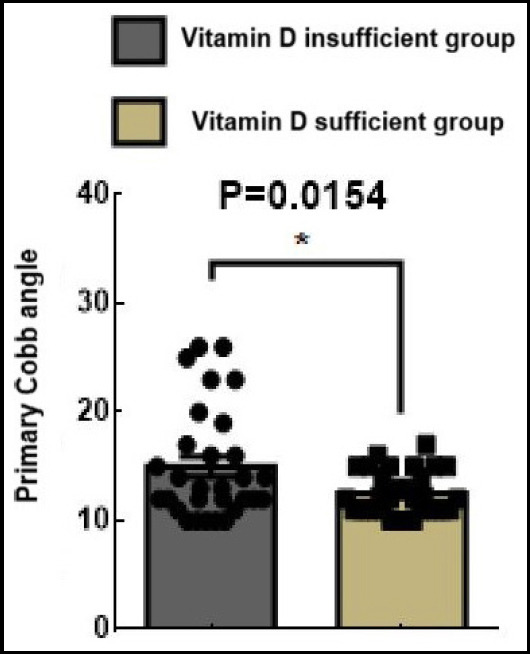
Correlation between Serum 25(OH)D Level and Cobb’s Angle in Patients with AIS.

Among patients with Vitamin-D deficiency or insufficiency (25[OH]D < 50 nmol/L), 11 exhibited a Cobb angle > 15°, whereas only two patients in the Vitamin-D sufficient group (25[OH]D > 50–250 nmol/L) had a Cobb angle > 15°. Statistical analysis was performed using Prism. Differences between groups were assessed using independent t-tests, and correlation analyses were conducted. A significant negative correlation was observed between serum 25(OH)D levels and the Cobb angle (*P =* 0.0154 < 0.05) (Fig.1).

## DISCUSSION

In the present study, patients with serum 25(OH)D levels below 50 nmol/L exhibited significantly greater Cobb angles compared with those with sufficient Vitamin-D levels. The observed negative correlation between serum Vitamin-D concentration and scoliosis severity(*P<* 0.05) supports previous reports suggesting that Cobb angle magnitude may be inversely associated with Vitamin-D status in patients with AIS. Furthermore, our findings are consistent with the hypothesis that Vitamin-D deficiency is more common in patients with AIS compared with the general healthy population, aligning with the known physiological functions of Vitamin-D. The *Practical Guidelines for Clinical Issues Related to Vitamin-D Nutrition in Chinese Children* also emphasize that the biological role of Vitamin-D extends beyond skeletal health to multiple systemic functions. Vitamin-D has been established to act not only through endocrine pathways but also via autocrine and paracrine mechanisms, and is therefore regarded as a hormone.^11^ The *2024* *Consensus on Diagnosis and Treatment of Adolescent Idiopathic Scoliosis*^15^ highlights that the pathogenesis of AIS is multifactorial, with substantial evidence implicating imbalanced growth and development, hormonal dysregulation (including estrogen, melatonin, and calmodulin)^16^, connective tissue abnormalities, and central nervous system dysfunction.^17^

Silva RTE et al.^18^ reported that Vitamin-D deficiency may be associated with the onset and progression of AIS, with patients showing significantly lower Vitamin-D levels compared with healthy controls. Similarly, Balioglu MB et al.^19^ in a cohort study of 229 patients with AIS, found that Vitamin-D levels were lower in patients with AIS than in controls and observed a negative correlation between Vitamin-D levels and Cobb angle magnitude. These findings are consistent with the results of our study, suggesting that Vitamin-D deficiency may play a role in curve progression. However, the precise mechanisms by which Vitamin-D influences AIS remain unclear and may involve interactions with other hormones and metabolic pathways in the central nervous system.

The peak onset of AIS occurs between 10 and 18 years of age, particularly during the pubertal growth spurt, and the condition is generally more prevalent in females than in males.^20^ In early childhood, Vitamin-D plays a critical role in skeletal growth and development, and inadequate Vitamin-D intake may adversely affect bone health in children and adolescents. Moreover, skeletal muscle requires sufficient Vitamin-D to maintain structural integrity and optimal function. Vitamin-D deficiency has been associated with reduced muscle mass and atrophy of Type-II muscle fibers.^11^ Before skeletal maturity, most AIS patients experience varying degrees of curve progression. Risk factors for progression are diverse, ranging from individual biological characteristics to radiographic parameters, and they are closely related to treatment and management strategies. Therefore, continuous, individualized, and standardized care throughout the disease course is essential to limit curve progression and optimize correction outcomes.

### Limitations:

First, the sample size was relatively small. Second, the study parameters were limited, focusing primarily on serum Vitamin-D levels. Third, sex and other demographic factors were not considered in the analysis. Finally, no mechanistic investigations were performed to clarify the role of Vitamin-D in AIS pathophysiology. Mechanistic studies are warranted to clarify the specific role of Vitamin-D in AIS.

## CONCLUSIONS

Vitamin-D deficiency may be associated with the development of adolescent idiopathic scoliosis. Adequate Vitamin-D supplementation could help mitigate curve progression and reduce scoliosis severity.

### Authors’ Contributions:

**XW** and **LJ:** Carried out the studies, drafted the manuscript, are responsible and accountable for the accuracy or integrity of the work.

**XW:** Collected the data and performed the analysis.

**JZ:** Writing of the manuscript and is responsible for the integrity of the study.

All authors read and approved the final manuscript.
